# The Traditional Herbal Medicine, Dangkwisoo-San, Prevents Cerebral Ischemic Injury through Nitric Oxide-Dependent Mechanisms

**DOI:** 10.1155/2011/718302

**Published:** 2011-02-23

**Authors:** Ji Hyun Kim, Sun Haeng Park, Young Whan Kim, Jung Min Ha, Sun Sik Bae, Guem San Lee, Su In Cho, Byung Tae Choi, Hwa Kyoung Shin

**Affiliations:** ^1^Division of Meridian and Structural Medicine, School of Korean Medicine, Pusan National University, Yangsan 626-870, Republic of Korea; ^2^Department of Pharmacology, School of Medicine, Pusan National University, Yangsan 626-870, Republic of Korea; ^3^Division of Pharmacology, School of Korean Medicine, Pusan National University, Yangsan 626-870, Republic of Korea

## Abstract

Dangkwisoo-San (DS) is an herbal extract that is widely used in traditional Korean medicine to treat traumatic ecchymosis and pain by promoting blood circulation and relieving blood stasis. However, the effect of DS in cerebrovascular disease has not been examined experimentally. The protective effects of DS on focal ischemic brain were investigated in a mouse model. DS stimulated nitric oxide (NO) production in human brain microvascular endothelial cells (HBMECs). DS (10–300 *μ*g/mL) produced a concentration-dependent relaxation in mouse aorta, which was significantly attenuated by the nitric oxide synthase (NOS) inhibitor L-NAME, suggesting that DS causes vasodilation via a NO-dependent mechanism. DS increased resting cerebral blood flow (CBF), although it caused mild hypotension. To investigate the effect of DS on the acute cerebral injury, C57/BL6J mice received 90 min of middle cerebral artery occlusion followed by 22.5 h of reperfusion. DS administered 3 days before arterial occlusion significantly reduced cerebral infarct size by 53.7% compared with vehicle treatment. However, DS did not reduce brain infarction in mice treated with the relatively specific endothelial NOS (eNOS) inhibitor, N^5^-(1-iminoethyl)-L-ornithine, suggesting that the neuroprotective effect of DS is primarily endothelium-dependent. This correlated with increased phosphorylation of eNOS in the brains of DS-treated mice. DS acutely improves CBF in eNOS-dependent vasodilation and reduces infarct size in focal cerebral ischemia. These data provide causal evidence that DS is cerebroprotective via the eNOS-dependent production of NO, which ameliorates blood circulation.

## 1. Introduction

Stroke is the main cause of adult disability and the third leading cause of death in the world [1]. Despite decades of intense research, the treatment of acute stroke remains limited. Therapies that restore cerebral blood flow (CBF) are efficacious in acute stroke, suggesting that CBF is a critical determinant of final stroke outcome. Endothelium-derived nitric oxide (NO) regulates CBF and mediates vascular response and prevents ischemic stroke by increasing collateral flow to the ischemic area [2, 3]. Thus, conditions that enhance endothelial NO synthase (eNOS) activity could have beneficial effects on stroke.

Herbal medicine may be useful for the treatment of stroke [4]. Traditional Korean medicine is based on natural plants and has many herbal prescriptions for treating stroke, but its therapeutic efficacies as well as its mechanisms are unclear. Dangkwisoo-San (DS) is used in traditional Korean medicine for the treatment of traumatic ecchymosis and pain by promoting blood circulation and relieving blood stasis. 

Traditional Korean medications usually contain many compounds that affect multiple targets [5, 6]. The combination of multiple drugs is thought to maximize therapeutic efficacy by facilitating synergistic actions and preventing potential adverse effects. DS contains nine species of herbal plants (Angelicae gigantis Radix, Paeoniae Radix, Linderae Radix, Sappan Lignum, Cyperi Rhizoma, Carthami Flos, Persicae Semen, Cinnamomi Cortex, and Glycyrrhizae Radix et Rhizoma) that have various pharmacological effects on the cardiovascular system [7–9]. However, no report has described the effects of DS on stroke in an animal model.

The present study examined the effects of DS on cerebral infarct, blood flow, blood pressure, and eNOS signaling in response to ischemia. To determine the physiological relevance of eNOS regulation by DS, DS was administered to control and N^5^-(1-iminoethyl)-L-ornithine (L-NIO)-treated mice for 3 days before subjecting them to middle cerebral artery occlusion. The findings suggest that DS has vascular protective action for acute cerebral ischemic damage through an eNOS-dependent mechanism.

## 2. Methods

### 2.1. Preparation of DS Extract

DS was purchased from Kwangmyungdang Natural Pharmaceutical (Ulsan, Korea). The herbal components were identified by one of the authors (Su In Cho). The mixture (60 g) of the nine constituent dried plants (Table 1) was boiled in 1 L of distilled water using an herb extractor (Dae-Woong, Korea) for 2 h. The final extract volume of 200 mL was centrifuged, and the supernatant was evaporated under reduced pressure at low temperature and lyophilized. The yield of the final products was 4.6 g.

### 2.2. Determination of NO Production in Human Brain Microvascular Endothelial Cells (HBMECs)

A membrane-permeable fluorescent indicator for NO (4-amino-5-methylamino-2′,7′-difluorofluorescein diacetate; DAF-FM; Molecular Probes, Eugene, OR) was utilized to detect DS-induced changes of the NO production. DAF-FM DA is converted via a NO-specific mechanism to an intensely fluorescent triazole derivative [10]. HBMECs obtained from Applied Cell Biology Research Institute (Kirkland, WA) were cultured in endothelial growth medium-2 (EGM-2) using a MV Bullet Kit system (Cambrex, Walkersville, MD). Experiments were performed after 4–6 cell passages. After reaching subfluency, the cells were incubated in endothelial cell basal medium (EBM, Cambrex) with DS and L-arginine for 10 min, and 5 *μ*mol/L DAF-FM DA was loaded into the cells. After incubation at 37°C for 5 min, the cells were mildly washed twice using phosphate-buffered saline (PBS) to eliminate any interference. The NOS inhibitor, N^G^-nitro-L-arginine methyl ester (L-NAME; 100 *μ*mol/L) and acetylcholine (30 *μ*mol/L) were used to validate the measurements. Fluorescence was detected using an Axiovert 200 fluorescence microscope (Carl Zeiss, Oberkochen, Germany).

### 2.3. Isolated Vessel Experiments

Male C57BL/6J mice were killed by decapitation. The thoracic aorta was removed and immersed in ice-cold modified Krebs' solution (composition, mmol/L: NaCl, 119.0; KCl, 4.7; NaHCO_3_, 20.0; MgSO_4_·7H_2_O, 1.2; KH_2_PO_4_, 1.2; CaCl_2_·2H_2_O, 2.5; glucose, 11; EDTA, 0.027; pH 7.4). The vessels were dissected for fat and connective tissues and cut into 1.5–2 mm long segments. The aortic rings were suspended at a tension of 1.3 g by means of two L-shaped stainless steel wires inserted into the lumen in 10 mL organ chambers filled with Krebs' solution maintained at 37°C and aerated continuously with 95% O_2_ and 5% CO_2_. The aortic rings were allowed to equilibrate for 90 min while changing the chamber solution every 15 min. After the resting tension of each vascular specimen was stabilized, sustained and stable contraction of 1.3 ± 0.6 g was maintained by adding phenylephrine (1 *μ*moL). The vasodilatory effect of DS was studied by cumulative addition of 10, 30, 100, and 300 *μ*g/mL DS at the plateau of the phenylephrine-induced contraction. Finally, endothelial-dependent relaxation was tested by subsequent application of acetylcholine (1 *μ*moL). Changes in isometric tension were computationally recorded using a model FT 0.3 force-displacement transducer (Grass, Quincy, MA) connected to a PowerLab ML 118 system 400 (AD Instruments, Medford, MA). Relaxations were expressed as the percentage of relaxation of phenylephrine-induced tone.

### 2.4. General Surgical Preparation

Male C57BL/6J mice weighing 20–25 g were housed under diurnal lighting conditions and allowed food and tap water *ad libitum*. All animal procedures were in accordance with the institutional guidelines for animal research and were approved by the Institutional Animal Care and Use Committee. Anesthesia was achieved by face mask-delivered isoflurane (2% induction and 1.5% maintenance in 70% nitrous oxide and 30% O_2_). The carotid artery and femoral vein were catheterized for the measurement of mean arterial blood pressure using a MLT844 physiological pressure transducer (AD Instruments) and the infusion of DS or saline. The depth of anesthesia was checked by the absence of cardiovascular changes in response to a tail pinch. Rectal temperature was kept at 36.5–37.5°C using a Panlab thermostatically controlled heating mat (Harvard Apparatus, Holliston, MA). The data were continuously recorded using a PowerLab data acquisition and analysis system (AD Instruments) and were stored in a computer. Mean arterial blood pressure, arterial blood gases, and pH were measured after a 3-day treatment of DS (i-Stat System, Abbott Laboratories, Abbott Park, IL). The physiological parameters were within the normal limits (Table 2).

### 2.5. Focal Cerebral Ischemia

Mice were orally administered with DS (600 mg/kg twice per day for 3 days) or saline before the ischemic insult. Focal cerebral ischemia was induced by occluding the middle cerebral artery (MCA) by the intraluminal filament technique [11, 12]. MCA occlusion (MCAO) was induced by the advancement of a silicon-coated 7–0 monofilament in the internal carotid artery. In all animals, regional cerebral blood flow (rCBF) was measured using a PeriFlux Laser Doppler System 5000 (Perimed, Stockholm, Sweden) with a flexible probe to confirm the achievement of consistent and similar levels of ischemic induction. The filament was withdrawn 90 min after occlusion, and reperfusion was confirmed using the laser Doppler apparatus. The surgical wound was sutured, and mice were allowed to recover from anesthesia. Brains were removed 24 h after MCA occlusion. Cerebral infarct size was determined on 2,3,5-triphenyltetrazolium chloride- (TTC-) stained, 2-mm-thick brain sections. Infarction areas were quantified with iSolution full image analysis software (Image & Microscope Technology, Vancouver, Canada). To account for and eliminate the effects of swelling/edema, infarction volume was calculated by an indirect measurement by summing the volumes of each section according to the following formula: contralateral hemisphere (mm^3^) − undamaged ipsilateral hemisphere (mm^3^).

### 2.6. Rotarod Test

Rotarod has been used to evaluate motor coordination by testing the ability of mice to remain on revolving rod [13]. The apparatus consisted of horizontal rough metal rod of 3 cm diameter attached to a motor with variable speed (Neo Click Co., kyeonggi-do, Korea). The rate of rotation was adjusted to allow the normal mice to stay on it for 5 min. Each mouse was given five trials before the actual reading was taken. The animals staying on revolving rod for period of 5 min before the surgical procedure were selected, and the test was again performed after focal cerebral ischemia followed by 24 h reperfusion.

### 2.7. Western Blotting

Brain tissues were collected 90 min after induction of ischemia. Proteins were isolated according to standard techniques, separated by 10% sodium dodecyl sulfate-polyacrylamide gel electrophoresis, and transferred onto a nitrocellulose membrane (Amersham Biosciences, Piscataway, NJ). Immunoblot analysis was performed with anti-eNOS and anti-phospho-eNOS (pS1177) antibodies (BD Biosciences, San Jose, CA), anti-Akt and anti-phospho-Akt (Ser 473) antibodies (Cell signaling, Danvers, MA), anti-nNOS and anti-iNOS antibodies (Santa Cruz Biotechnology, Santa Cruz, CA) followed by incubation with secondary antibody conjugated with horseradish peroxidase. The intensity of chemiluminescence was measured using an ImageQuant LAS 4000 apparatus (GE Healthcare Life Sciences, Uppsala, Sweden). The membrane was reprobed with an anti-*β*-actin antibody (Sigma-Aldrich, St. Louis, MO) as an internal control.

### 2.8. Chemicals

Acetylcholine chloride, L-NAME, and phenylephrine hydrochloride were purchased from Sigma-Aldrich. L-NIO was purchased from Tocris Bioscience (Bristol, UK), and all other chemicals were reagent grade. The solid form of the extract was dissolved in distilled water.

### 2.9. Data Analysis

The data were expressed as mean ± SEM. Statistical comparisons were performed using paired or unpaired Student's *t*-test and one-way analysis of variance (ANOVA) or two-way ANOVA for repeated measures followed by Fisher's protected least significant difference test. *P* < .05 was considered statistically significant.

## 3. Results

### 3.1. DS Stimulates NO Production in HBMEC

**Figure 1:**
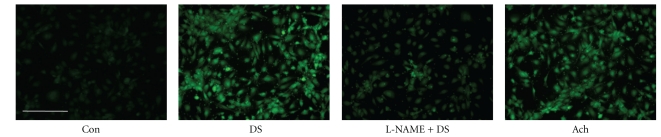
Effects of DS on NO production in HBMEC. HBMECs were loaded DAF-FM (50 *μ*mol/L) with DS (30 *μ*g/mL), in the absence or presence of the NOS inhibitor, L-NAME (100 *μ*mol/L). Acetylcholine (Ach, 30 *μ*mol/L) was used to validate our measurements. DS increased NO production, which was abrogated by L-NAME. The scale bar represents 200 *μ*m.

DAF-FM DA, a fluorescent NO-sensitive dye, was used to measure DS-induced NO release in HBMECs. The effect of the NOS inhibitor, L-NAME, was also assessed to determine whether the NO increase was attributable to NOS activity-derived *de novo* synthesis. NO production was rapid, being observed within 5 min of 30 *μ*g/mL DS addition, and reached a maximum at 10 min in HBMECs (data not shown). Treatment of HBMECs with 30 *μ*g/mL DS for 10 min induced NO production, which was abrogated by pretreatment with 100 *μ*mol/L L-NAME (Figure 1). Similar results were obtained in endothelial cells from mouse thoracic aorta (data not shown). This result was consistent with the suggestion that the increase in NO production after DS treatment was mediated by increased NOS activity.

### 3.2. DS Causes Vasorelaxation in Isolated Mouse Aorta

**Figure 2:**
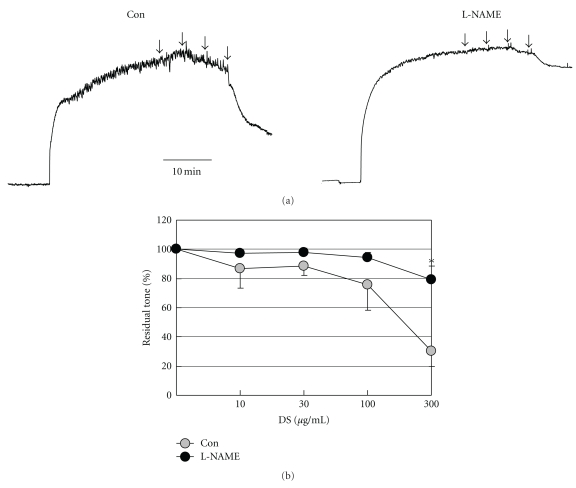
Effects of DS on phenylephrine-induced aortic contraction. (a) Representative tracings showing that cumulative addition of DS (10, 30, 100, and 300 *μ*g/mL; arrows) relaxed mouse aorta in a concentration-dependent manner in vessel segments preconstricted with phenylephrine (PE, 1 *μ*mol/L). (b) Average of data from four preparations showing that incubation with 100 *μ*mol/L L-NAME abolished the DS-mediated relaxation. *P < .05 versus control, two-way ANOVA for repeated measures. Error bars indicate standard errors and are shown unidirectionally for clarity.

DS concentrations of 10–300 *μ*g/mL relaxed isolated mouse aorta in a concentration-dependent manner with a maximum value of 30.02 ± 10.39% at a concentration of 300 *μ*g/mL. This relaxation was abolished by the NOS inhibitor L-NAME (100 *μ*mol/L) (Figure 2), confirming the NO-mediated nature.

### 3.3. Effects of DS on Blood Pressure and Resting CBF

**Figure 3:**
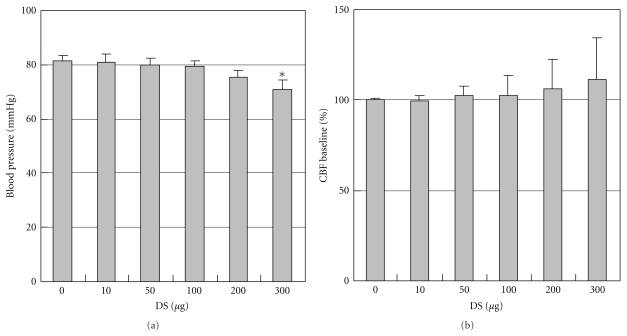
Effects of DS on blood pressure and resting CBF. Mean arterial blood pressure (MABP) was measured from the femoral artery (a) and cerebral blood flow (CBF) was measured within middle cerebral artery branches (b) during intravenous infusion of DS. Values are expressed as a percent change from baseline immediately before infusion. DS slightly increased resting CBF in a concentration-dependent manner in the cortex (*P* > .05 versus baseline), although it caused mild hypotension. Values are expressed as means ± SEM of five separate experiments. *P < .05 versus control.

DS caused mild hypotension (81.5 ± 2.1 mmHg versus 71 ± 3.2 mmHg, in control and 300 *μ*g/kg DS group; *P* < .05, *N* = 5; Figure 3(a)) at a concentration of 300 *μ*g/kg when infused via mouse femoral vein. Resting CBF was increased in the cortex in mice treated with 100–300 *μ*g/kg DS in a concentration-dependent manner, even DS caused mild hypotension (Figure 3(b)).

### 3.4. Protective Actions of DS on Cerebral Ischemic Injury

**Figure 4:**
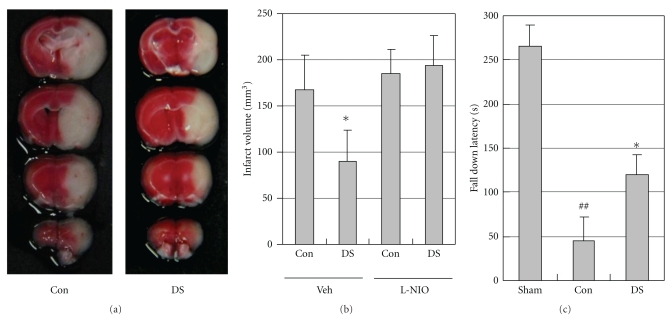
DS reduces cerebral ischemic injury. (a) Representative photographs of coronal brain sections stained with 2,3,5-triphenyltetrazolium chloride in saline- (Con, left)—and DS-treated mice (right). Mice were orally administered saline or 600 mg/kg DS twice per day for 3 days before the ischemic insult. Mice were subjected to 90 min of MCAO followed by 22.5 h of reperfusion. White indicates the infarct area. (b) Effect of DS on infarct volume in saline- and L-NIO-treated mice at 24 h after ischemia. Infarction volume was calculated by an indirect measurement. DS significantly reduced cerebral infarct size; however, it did not affect brain infarction in L-NIO-treated mice. (c) Effect of DS on ischemia and reperfusion induced impairment of motor coordination. DS markedly prevented ischemia-reperfusion induced motor incoordination. Data are expressed as means ± SEM of six separate experiments. *P < .05 versus control; ^##^ *P* < .01 versus Sham group.

To determine whether DS could protect against ischemic stroke, DS was administered to mice for 3 days before MCAO. DS decreased cerebral infarct volume (167.0 ± 38.2 mm^3^) as compared with vehicle treatment (89.7 ± 34.0 mm^3^; *P* < .05, *N* = 6; Figures 4(a) and 4(b)). Focal cerebral ischemia followed by reperfusion produced significant motor incoordination (*P* < .01, *N* = 5) in mice measured by rotarod test as compared to that of sham group animals. DS markedly prevented ischemia-reperfusion induced motor incoordination (*P* < .05, *N* = 5; Figure 4(c)). To examine the contribution of eNOS signaling to the cerebroprotective action of DS, an experiment tested the impact of DS on ischemic injury in mice treated with the relatively specific eNOS inhibitor, L-NIO. In contrast to control, DS treatment failed to reduce infarct volume in L-NIO-treated mice (Figure 4(b)).

### 3.5. DS Protects against Ischemic Stroke through eNOS-Dependent Signaling

**Figure 5:**
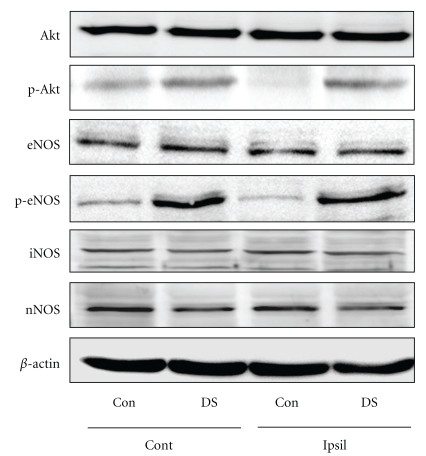
Effects of DS on phosphorylation of Akt and eNOS in brain tissues. Phosphorylation of Akt (p-Akt) and eNOS (p-eNOS) in brain tissues of saline- (Con) and DS-treated mice at 60 min after ischemia. Akt, p-Akt, eNOS, p-eNOS, iNOS, and nNOS protein levels were analyzed by Western blotting (*N* = 4). DS promoted Akt and eNOS phosphorylation in both ischemic (ipsilateral, Ipsil) and nonischemic regions (contralateral, Cont) of the brain compared with control.

To further assess the impact of DS on eNOS signaling during ischemia, the phosphorylation of Akt at Ser473 and eNOS at Ser1177 in brain tissues was assessed by Western blotting. DS treatment promoted Akt and eNOS phosphorylation in both ischemic and nonischemic regions of the brain compared with control. However, total Akt, eNOS, iNOS, and nNOS protein levels did not differ between the DS-treated and control mice (Figure 5).

## 4. Discussion

DS, an herbal extract, is widely used in traditional Korean medicine to treat traumatic ecchymosis and pain by promoting blood circulation and relieving blood stasis. However, the effect of DS in cerebrovascular disease has not been examined experimentally. The present study provides evidence that DS protects the brain from acute ischemic injury in a mouse model of MCAO. DS increased NO production, which led to vasodilation, improved CBF, and decreased cerebral infarction size. The cerebroprotective effect of DS was mediated by eNOS, given that DS had no beneficial effect on cerebral infarction size in mice treated with L-NIO. Indeed, phosphorylated eNOS was increased in brain tissue after DS treatment. The present observations indicate that DS exerts a cerebroprotective action through an eNOS-dependent mechanism.

When DS was administered 3 days before subjecting mice to MCAO, cerebral infarct volume was significantly decreased (Figure 4). However, it is not known whether this was due to a vasodilator effect on cerebral vessels leading to an acute augmentation of CBF or other mechanisms such as a direct neuroprotective action [14, 15]. Presently, DS caused vasodilation and improved CBF, even it caused mild hypotension. The endothelial and smooth muscle mechanisms of vasodilation mediated by DS appear to be differentially active in systemic and cerebral circulation. Therefore, the direct smooth muscle relaxant effect of DS mildly decreased systemic resistance, but not cerebrovascular resistance. In light of the potential detrimental effect of systemic vasodilation and hyperemia on CBF in acute stroke, DS may be more efficacious in stroke therapy. 

Therapies that restore CBF to ischemic regions are efficacious in acute stroke, suggesting that CBF is a critical determinant of stroke outcome. NO constitutively produced by eNOS regulates CBF and mediates vascular response and protects against ischemic stroke by mediating vasodilation and hence increases blood flow to the damaged brain area [3, 16, 17]. Several lines of evidence indicate that NO donor or L-arginine improves blood flow and reduces tissue damage after focal cerebral ischemia [18, 19]. Several therapeutic modalities to upregulate and/or active eNOS might mediate NO-dependent stroke-protective effects [20]. The beneficial effects of DS on ischemic injury are due at least in part to its vascular protective actions, which involve eNOS-dependent mechanisms, because the cerebroprotective actions of DS are abolished in a relatively specific eNOS inhibitor [21], L-NIO-treated mice. Consistent with these findings, Akt and eNOS phosphorylation was presently increased in the brains of DS-treated mice. Endothelial NO release is enhanced through direct phosphorylation of eNOS by the protein kinase Akt downstream of PI3K [22]. In contrast, total Akt, eNOS, iNOS, and nNOS expression level did not differ between vehicle- and DS-treated mice. Together, the present results clearly demonstrate that DS regulates eNOS signaling to modulate vascular function under ischemic conditions, protecting against cerebral injury after stroke.

DS represents a mixture of nine herbal medicines, consisting of Angelicae gigantis Radix, Paeoniae Radix, Linderae Radix, Sappan Lignum, Cyperi Rhizoma, Carthami Flos, Persicae Semen, Cinnamomi Cortex, and Glycyrrhizae Radix et Rhizoma. Most traditional therapeutic formulations consist of a combination of several drugs. Bioactivity from each drug may collectively act to block multiple targets underlying ischemic pathophysiology, although little is known about the mechanisms for their pharmacological activities [5, 6]. The combination of multiple drugs is thought to maximize therapeutic efficacy by facilitating synergistic actions and preventing potential adverse effects. However, little is known concerning the compounds responsible for the protective effect of DS. It will be important to perform additional experiments to identify the efficient compounds from DS. 

In summary, DS increases NO production, vasodilation and improvement of CBF, which protects against cerebral ischemia. The DS-mediated cerebroprotective effects are absent in mice treated with a relatively specific eNOS inhibitor, L-NIO, and eNOS phosphorylation is increased in the brains of mice treated with DS, indicating the obligatory role of endothelium-derived NO in mediating these beneficial effects. 

## Figures and Tables

**Table 1:** Composition of Dangkwisoo-San.

Scientific name	Herbal name	Amount (g)
*Angelica gigas *Nakai	Angelicae gigantis Radix	5.625
*Paeonia lactiflora *Pall	Paeoniae Radix	3.750
*Lindera strichnifolia *Fernández-Villar	Linderae Radix	3.750
*Caesalpinia sappan *L.	Sappan Lignum	3.750
*Cyperus rotundus *L.	Cyperi Rhizoma	3.750
*Carthamus tinctorious *L.	Carthami Flos	3.000
*Prunus persica *Batsch	Persicae Semen	2.655
*Cinnamomum cassia *Presl	Cinnamomi Cortex	2.250
*Glycyrrhiza uralensis *Fisch	Glycyrrhizae Radix et Rhizoma	1.875
	Total	30.405

**Table 2:** Physiological parameters.

	Control (*N* = 7)	DS (*N* = 7)
MABP	80.6 ± 2.2	84.2 ± 2.0
pH	7.32 ± 0.01	7.36 ± 0.04
pCO_2_	41.1 ± 2.0	39.4 ± 2.5
pO_2_	143.8 ± 10.5	148.0 ± 6.0

Values are mean ± SEM. MABP (mean arterial blood pressure), pO_2_, and pCO_2_ are expressed in mmHg.
